# 快速溶剂萃取-离子色谱-质谱法测定人体血液、尿液中的氟乙酸

**DOI:** 10.3724/SP.J.1123.2022.09019

**Published:** 2023-06-08

**Authors:** Yuheng WANG, Jingwen ZHANG, Hongguo ZHENG, Sijia LU, Suhua YU, Ruiqin YANG, Yong WANG

**Affiliations:** 1.中国人民公安大学侦查学院, 北京 100038; 1. School of Investigation, People’s Public Security University of China, Beijing 100038, China; 2.赛默飞世尔科技(中国)有限公司, 四川 成都 610023; 2. Thermo Fisher Scientific Inc., Chengdu 610023, China; 3.南京市公安局刑事科学技术研究所, 江苏 南京 210001; 3. Institute of Forensic Science and Technology of Nanjing Public Security Bureau, Nanjing 210001, China

**Keywords:** 快速溶剂萃取, 离心超滤, 离子色谱-串联质谱法, 氟乙酸, 血液, 尿液, accelerated solvent extraction (ASE), centrifugal ultrafiltration, ion chromatography-tandem mass spectrometry (IC-MS/MS), fluoroacetic acid, blood, urine

## Abstract

建立了快速溶剂萃取-离子色谱-质谱法测定人体血液、尿液中氟乙酸的方法。以去离子水为萃取溶剂,使用快速溶剂萃取仪处理血液和尿液样品,取上清液依次经超滤管和0.22 μm水相针式滤膜净化,稀释50倍后进样检测。采用Ion Pac AS20离子色谱柱以15.0 mmol/L的KOH溶液为淋洗液进行等度淋洗,流出液通过抑制器后进入三重四极杆质谱,在负离子、多反应监测模式下检测,外标法定量。结果表明,氟乙酸在0.5~500.0 μg/L范围内线性关系良好(*r*>0.999),检出限和定量限分别为0.14、0.47 μg/L。氟乙酸在血液和尿液中的回收率分别为93.4%~95.8%、96.2%~98.4%,日内精密度分别为0.8%~1.6%、0.2%~1.0%,日间精密度分别为2.3%~3.8%、3.9%~6.9%。进一步考察发现该方法在血液、尿液中的基质效应较弱,分别为-7.4%、-3.0%。该法无需衍生化处理,简便高效,灵敏度高,重复性好,适用于人体血液、尿液中氟乙酸的快速检测。

## Abstract (en)

Fluoroacetic acid is a highly polar poison used for rodent control. When ingested by the human body, it seriously damages nerve cells and heart tissues and even causes death by cardiac arrest or respiratory failure. Common detection methods for fluoroacetic acid include gas chromatography-mass spectrometry and liquid chromatography-mass spectrometry, both of which require complex pretreatment methods, such as derivatization. In this study, a method to determine fluoroacetic acid in human blood and urine based on accelerated solvent extraction-ion chromatography-mass spectrometry (ASE-IC-MS) was established. Two pretreatment methods, namely, acetonitrile precipitation and accelerated solvent extraction, were compared. Furthermore, the effects of different extraction conditions, such as the extraction time, extraction temperature, and number of cycles, were investigated. The most suitable chromatographic separation conditions, such as the chromatographic column, column temperature, and elution procedure, were determined, and the MS conditions, such as the collision energy (CE) and declustering potential (DP) of the ion pairs of the target compound, were investigated. Based on the experimental results, the optimal pretreatment methods and detection conditions were obtained, and reliable data were collected. Deionized water was used as the extraction solvent, and blood and urine samples were processed by accelerated solvent extractor. The supernatant was sequentially collected via centrifugal ultrafiltration and 0.22 μm membrane filtration, diluted 50 times, and then injected into the chromatographic column for detection. An Ion Pac AS20 IC column was used for isocratic elution with 15.0 mmol/L KOH solution as the eluent. The effluent was passed through a suppressor and into a triple quadrupole mass spectrometer, which was used to perform MS/MS (ESI^-^) in multiple reaction monitoring (MRM) mode. The quantitative ion was *m/z* 77.0>57.0 when the CE and DP were -15.0 eV and -20.0 V, respectively. An external standard method was used for quantitative analysis. The results showed a good linear relationship for fluoroacetic acid in the range of 0.5-500.0 μg/L (*r*>0.999), with limits of detection (LOD) and quantification (LOQ) of 0.14 and 0.47 μg/L, respectively. The recoveries of fluoroacetic acid in blood and urine were 93.4%-95.8% and 96.2%-98.4%, respectively. The intra-day RSDs for blood and urine were 0.8%-1.6% and 0.2%-1.0%, respectively, while the inter-day RSDs were 2.3%-3.8% and 3.9%-6.9%, respectively. Further investigation revealed that the matrix effects of this method in blood and urine, at -7.4% and -3.0%, respectively, were fairly weak. The established method was successfully applied to detect fluoroacetic acid in human blood and urine obtained from a poisoning case, and the results obtained provided crucial clues that led to swift case resolution. The efficiency of the method was significantly higher than that of conventional detection methods. In conclusion, the developed method has high sensitivity and good repeatability and is suitable for the rapid detection of fluoroacetic acid in human blood and urine. Moreover, because this method does not require derivatization, it is simple and efficient.

氟乙酸是一种用于杀灭啮齿类动物的强极性毒物,主要用于灭鼠工作,大鼠经口半数致死量为0.22 mg/kg。该种毒物对动物和人体均有剧毒,被人体摄入后会严重损害神经细胞和心脏等组织,造成心脏骤停或呼吸衰竭而死亡^[1]^。因氟乙酸毒性强^[2]^,且具有二次毒性^[3]^, 1982年被我国禁止生产、销售和使用,但由于其杀鼠效果好,社会上违规生产此类毒物的现象屡见不鲜,进而导致大量误食或投毒事件的发生。能否在血液、尿液等生物检材中高效、准确地检测氟乙酸,一定程度上成为分析中毒案件中毒物的关键^[4]^,从而为患者的救助或死者死因的确定提供充足依据。但现有研究一方面在检测方法上存在前处理复杂、灵敏度低等问题,另一方面主要针对食物等基质^[5]^,对血液和尿液等复杂生物基质的研究不充分。综上所述,建立更加完备的人体血液、尿液等生物检材中氟乙酸的检测方法具有紧迫的现实需求。

针对血液、尿液等生物检材,已有研究大多采用乙腈或乙酸等有机溶剂沉淀蛋白质、固相萃取柱净化提取液的思路。该法相对成熟,但仍存在有机溶剂用量大、固相萃取柱活化时间长等缺点,而近年来在法庭科学和环境监测^[6]^等领域逐步得到运用的快速溶剂萃取法(ASE)则能够有效解决上述问题。已有研究表明,ASE能够通过提供高温、高压等仪器条件^[7]^,实现对固体或半固体类复杂基质中目标物的快速、高效萃取。在已有方法的基础上,应剑波等^[8]^以丙酮、二氯甲烷、环己烷混合溶剂为萃取液,利用ASE处理血液等复杂基质,大幅提高了目标物的萃取效率,将ASE的应用范围拓展至生物体液等液体基质。除了提高萃取效率,ASE高温、高压的动态萃取环境还能将血液、尿液等生物体液中的蛋白质充分变性,以达到沉淀蛋白质、释放目标物的效果,与离子色谱等检测手段结合可进一步实现非有机溶剂萃取和快速处理^[9]^等实验需求。ASE具有自动化程度高、方法便捷高效等优点,在处理血液、尿液等复杂生物检材方面有着广阔的应用前景。

氟乙酸在人体内主要以游离的氟乙酸根离子形式存在,因此检验此类鼠药的关键是检测氟乙酸根离子。目前,检测氟乙酸的方法有:气相色谱-质谱法(GC-MS)^[10,11]^、液相色谱-质谱法(LC-MS)^[12⇓-14]^和离子色谱法(IC)^[15]^等。其中,GC-MS需利用五氟苄基溴衍生化,并进行氮吹等复杂的前处理操作^[16]^;使用LC-MS检测时,由于氟乙酸极性强,C18等反相色谱柱无法直接分离,同样需要衍生化以降低极性;IC能够简便、高效地分离^[17]^氟乙酸等强极性物质^[18]^,但通过保留时间定性难以排除干扰离子的共存且灵敏度有待提高;三重四极杆质谱(MS/MS)通过离子对信息所反映的物质结构进行定性和定量分析,具有结果可靠、灵敏度高等优势。本研究使用三重四极杆质谱取代离子色谱的电导检测器,实现了离子色谱与质谱的联用,既结合了离子色谱高效分离极性物质的特点,又能发挥质谱定性准确^[19]^的优势,避免离子色谱检测时“假阳性”现象的发生。

一直以来,毒物检测是刑事技术工作的重点。本研究提出并验证了一种血液、尿液中氟乙酸的ASE前处理技术,弥补了刑事技术领域提取生物检材中氟乙酸的方法不足等问题,具有沉淀蛋白质效果好、萃取效率和自动化程度高、无须衍生化等显著优点;同时,建立了离子色谱-三重四极杆质谱(IC-MS/MS)测定人体血液、尿液中氟乙酸的检验方法,拓展了IC-MS/MS技术的应用场景,解决了既有检测技术操作复杂、灵敏度低等问题,为公安实战中氟乙酸的定性定量分析提供参考和依据。

## 1 实验部分

### 1.1 仪器、试剂与材料

ICS-5000+型离子色谱仪(美国赛默飞世尔公司):配电导检测器和ADRS-600 2 mm阴离子抑制器;QTRAP 4500三重四极杆质谱仪(美国Sciex公司); Milli-Q超纯水仪(美国密理博公司); ASE 350快速溶剂萃取仪(美国Dionex公司); Vortex-6快速混匀仪(山东博科控股集团有限公司); Centrifuge 5430台式高速离心机(德国Eppendorf公司); Ultra-4超滤管(3 kDa MWCO)(德国默克公司); 0.22 μm水相针式滤膜(浙江哈迈科技有限公司); Dionex OnGuardⅡ RP固相萃取柱(美国赛默飞世尔公司)。

氟乙酸标准溶液(1000 mg/L,北京芬格尔安科技有限责任公司),甲醇和乙腈(色谱纯,美国Supelco公司), 18.2 MΩ·cm去离子水由Milli-Q超纯水仪制得。

### 1.2 标准溶液配制

准确移取1000 mg/L的氟乙酸标准溶液,用去离子水稀释成10 mg/L的标准中间溶液,4 ℃下低温保存。根据实验需要,移取10 mg/L的标准溶液,用去离子水配制成系列浓度的标准工作溶液,现用现配。

### 1.3 样品提取和净化

将滤纸填入萃取池,在萃取池底部加入2 g硅藻土,取1 mL血液或尿液滴入后,在上层填入少量硅藻土。设置萃取温度120 ℃,置换率60%,加热6 min,静态萃取5 min,氮气吹扫100 s,循环次数2次,流速1 mL/min。

从ASE收集瓶中获取浸出液10 mL,静置10 min,取上清液2 mL于Ultra-4超滤管中离心8 min。离心后的滤液过0.22 μm水相针式滤膜并稀释50倍,得到待测液。

### 1.4 色谱条件

离子色谱柱:Ion Pac AS20 (250 mm×2 mm);保护柱:Ion Pac AG20 (50 mm×2 mm);抑制器:ADRS-600;淋洗液:KOH溶液;淋洗液浓度:15.0 mmol/L;进样量:25.0 μL;流速:0.25 mL/min;柱温:30 ℃。

### 1.5 质谱条件

离子源:电喷雾电离源,负离子模式(ESI^-^);检测模式:多反应监测(MRM);气帘气(CUR)压力:241.3 kPa;碰撞气强度(CAD): Medium;离子化电压(IS): -4500 V;离子源温度(TEM): 5500 ℃;喷雾气压(GS1): 379.2 kPa;辅助加热气压(GS2): 379.2 kPa。定量离子对为*m/z* 77.0>57.0,碰撞电压为-15.0 eV,去簇电压为-20.0 V。

## 2 结果与讨论

### 2.1 色谱条件优化

本研究依次对离子色谱柱和淋洗条件进行了考察。氟乙酸极性强,在水中呈离子形态,对阴离子交换固定相具有较强的亲和力,在常规阴离子交换柱上易出现峰展宽、峰拖尾、不易被洗脱等现象,因此需要对具有较强亲水性的不同色谱柱进行考察。对比了适用于强极性物质或有机酸分离的AS11-HC、AS19和AS20色谱柱。其中,AS11-HC色谱柱使用高交联度的乙基乙烯基苯-二乙烯基苯共聚物(EVB-DVB)为填料,适合分离复杂样品基质中的有机酸;AS19色谱柱采用超孔型EVB-DVB颗粒为主要填料,对各类卤氧化物和常见无机阴离子等强极性物质的分离效果较好;AS20色谱柱以EVB-DVB填料为基础,使用多层超支化季铵涂层,对高氯酸盐等强极性物质具有较好的分离效果,故这3种色谱柱理论上均能高效分离氟乙酸。实验结果(见图1)表明:AS11-HC色谱柱对氟乙酸的分离效果不佳,色谱峰拖尾严重;AS19色谱柱对氟乙酸的分离效果优于AS11-HC,但洗脱能力较差,存在峰展宽和峰拖尾的现象;而AS20色谱柱既能高效地分离氟乙酸,又能得到峰形较好的谱图,因此选用AS20色谱柱。

**图1:**
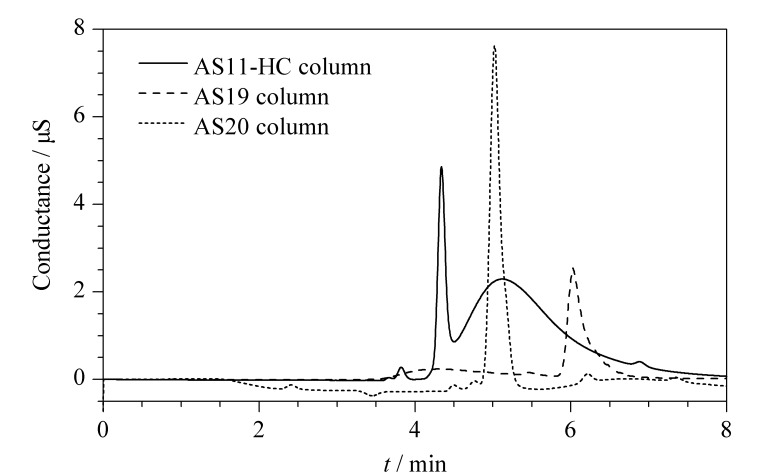
氟乙酸在不同柱上的色谱图

分别使用10.0、15.0、25.0 mmol/L的KOH溶液进行等度淋洗,结果表明:3种KOH浓度均能在较短时间内完成氟乙酸的分离,但15.0 mmol/L的KOH峰形较好、基线最稳定,且低浓度的洗脱条件有利于节省淋洗液,因此使用15.0 mmol/L的KOH溶液等度淋洗。

### 2.2 质谱条件优化

使用甲醇-水(1∶1, v/v)配制200 μg/L的氟乙酸工作溶液,采用流动注射方式进样,在电喷雾负离子模式下确定氟乙酸的质谱条件。Q1 MS模式扫描得到氟乙酸的母离子为*m/z* 77.0, MS^2^模式扫描得到子离子*m/z* 57.0和*m/z* 33.0, MRM模式优化母离子和子离子的碰撞能和去簇电压。综合考虑信噪比和碎片离子响应,选择子离子*m/z* 57.0为定量离子。

### 2.3 前处理条件优化

#### 2.3.1 前处理方法选择

血液和尿液等生物体液成分复杂,含有蛋白质等干扰物,净化不充分会严重影响目标物的检测效果,甚至污染分析仪器。乙腈沉淀蛋白质-RP固相萃取柱净化是离子色谱检测血液、尿液最常用的前处理方法,该法通过乙腈析出样品中的蛋白质、反相(RP)柱除去有机相的方式净化样品。实验结果表明:在以10.0、50.0、200.0 μg/L水平添加的条件下,氟乙酸在血液和尿液中的平均回收率(*n*=6)分别为106.7%~109.7%、107.2%~112.9%(见表1)。虽然该法能够较好地处理生物检材中的蛋白质,提高质谱响应,但仍存在残留有机溶剂增强基质效应、引起离子色谱柱损耗等问题。

**表1:** 不同净化方式下氟乙酸的提取回收率(*n*=6)

Method	Added/ (μg/L)	Found/(μg/L)			Recovery/%	
		Blood	Urine		Blood	Urine
Acetonitrile-	10.0	10.8	11.3		108.4	112.9
RP column	50.0	54.9	53.6		109.7	107.2
	200.0	213.3	221.7		106.7	110.9
ASE-centrifugal	10.0	9.5	9.6		94.8	96.2
ultrafiltration	50.0	46.7	48.9		93.4	97.9
	200.0	191.5	196.7		95.8	98.4

离子色谱柱和淋洗液的性质决定了水溶液为最佳前处理萃取溶剂,而使用水溶液直接萃取血液和尿液中的目标物主要存在两方面的问题,一是由于目标物会与血液、尿液中的成分结合,简单的液液萃取效率低,因此难以将目标物从检材中萃取出来;二是无法直接对蛋白质进行沉淀,萃取后的液体不能直接用于离子色谱的进样检测。综合考虑萃取溶剂、提取效率和操作简便程度,本研究选定高温变性沉淀蛋白质的原理探索前处理方法。以水为萃取溶剂的快速溶剂萃取仪能够提供稳定的高温、高压萃取条件,既避免了引入有机溶剂,又有利于血液和尿液中的蛋白质充分沉淀,同时极大地提高了目标物的萃取效率。此外,离心超滤法能有效净化样品提取液且不会引起氟乙酸的损耗。

考察了快速溶剂萃取-离心超滤法提取血液、尿液中氟乙酸的效果,试验表明:在以10.0、50.0、200.0 μg/L水平添加的条件下,氟乙酸在血液和尿液中的平均回收率分别为87.2%~93.5%、93.7%~98.1%。结果验证了该前处理方式的可行性与稳定性,即在不引入有机溶剂的情况下,快速溶剂萃取-离心超滤法沉淀蛋白质和净化样品的效果同样显著,且能保持较高的回收率。此外,由于RP柱活化时间长、活化过程复杂,而快速溶剂萃取仪可以批量进样、全自动处理,故ASE-离心超滤法操作还具有耗时短、操作简便等优势。综上所述,本研究采用ASE-离心超滤作为血液和尿液样品的前处理方法。

#### 2.3.2 温度对萃取效果的影响

温度是快速溶剂萃取效率的决定性因素,随着萃取温度升高,目标化合物的溶解性增强、扩散速度加快^[20]^,血液和尿液中蛋白质的变性也更充分。对空白血液、尿液进行标准样品添加试验,考察方法回收率受温度影响的程度。取空白检材,添加标准溶液,配制成氟乙酸质量浓度为1 mg/L的添加样品。

对比不同萃取温度(100、110、120、130、140 ℃)下氟乙酸的回收率和沉淀蛋白质的效果,结果表明:在100~120 ℃范围内,回收率随温度升高有明显的提高,大于120 ℃时,回收率维持稳定,结果见图2;萃取温度为100 ℃时,蛋白质沉淀效果差,样品无法充分净化,110~140 ℃范围内,蛋白质均充分沉淀;此外,温度越高所需升温时间越长。综合对比回收率、沉淀蛋白质效果和升温时间等因素,选择萃取温度为120 ℃。

**图2:**
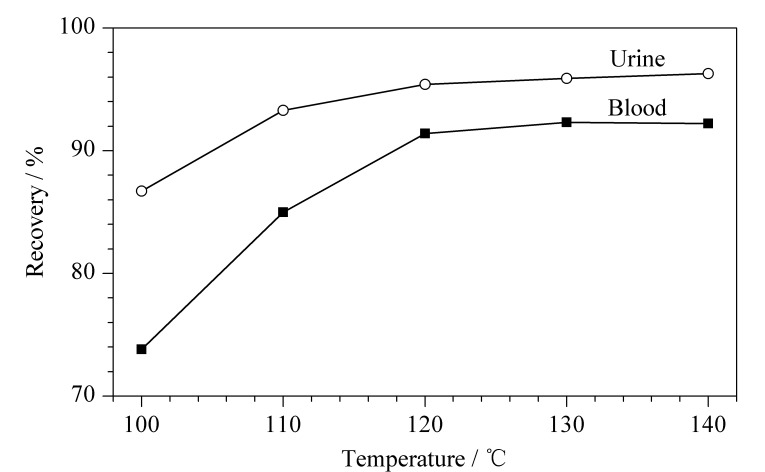
萃取温度对氟乙酸回收率的影响(*n*=3)

#### 2.3.3 萃取时间和循环次数的选择

静态萃取时间和循环次数直接影响目标物的萃取程度,延长萃取时间或增多循环次数可以获得更好的萃取效果,但也需要更长的前处理时间,因此需要选择合适的萃取时间和循环次数,以确保在实现高萃取效率的同时节省样品处理时间。保持2次循环,考察不同萃取时间(3、4、5、6、7 min)对萃取效率的影响,结果显示:3~5 min内萃取效率随时间增加而明显提高,5 min后萃取效率没有显著差异,见表2。这表明在2次循环的条件下,5 min的静态萃取时间足以使氟乙酸接近生物检材和溶剂之间的分配平衡。

**表2:** 不同快速溶剂萃取条件下氟乙酸的提取效率(*n*=3)

Matrix	Added/ (μg/L)	Recoveries under different cycle numbers×extraction times (min)/%							
		2×3	2×4	2×5	2×6	2×7	1×5	2×5	3×5
Blood	50.0	75.3	88.2	93.4	92.0	93.6	71.3	101.5	93.9
	200.0	74.7	90.0	95.8	95.9	96.1	74.0	95.8	96.8
Urine	50.0	81.4	94.1	97.9	97.8	96.9	78.1	97.9	98.4
	200.0	82.7	97.3	98.4	98.5	98.7	77.3	98.4	98.8

选定静态萃取5 min,考察1~3次循环对萃取效率的影响。在每次循环中,新溶剂进入萃取池,待测物在新溶剂与残留样品之间重新建立分配平衡,进而促进待测物被萃取剂充分提取。结果(见表2)表明,循环从1次增加到2次,氟乙酸的提取效率显著提高;但循环从2次增加到3次时,氟乙酸的提取效率仅得到微弱提高,说明循环进行到第2次时,待测物氟乙酸已经被充分回收。综上所述,循环2次、单次静态萃取5 min为最佳ASE条件。

### 2.4 方法学考察

#### 2.4.1 线性关系、检出限与定量限

使用10 mg/L的氟乙酸标准溶液配制成质量浓度分别为0.5、1.0、5.0、20.0、100.0、200.0、500.0 μg/L的系列浓度氟乙酸标准工作溶液,以标准工作溶液中氟乙酸的浓度(*x*, μg/L)为横坐标,以标准工作溶液中氟乙酸的定量离子峰面积(*y*)为纵坐标,绘制标准工作曲线。结果表明:氟乙酸在0.5~500.0 μg/L范围内线性关系良好,相关系数大于0.999,线性方程为*y*=15620.3*x*+3672.2;以信噪比(*S/N*)≥3确定检出限为0.14 μg/L,以*S/N*≥10确定定量限为0.47 μg/L。

#### 2.4.2 回收率与精密度

向空白血液和尿液中分别添加10.0、50.0、200.0 μg/L 3种不同浓度的氟乙酸标准工作溶液并混匀静置1 h。按1.3节的步骤处理上述样品,连续6 d每天测定6次,获得回收率和精密度数据。结果表明:氟乙酸在血液和尿液中的平均回收率分别为93.4%~95.8%和96.2%~98.4%,日内精密度分别为0.8%~1.6%、0.2%~1.0%,日间精密度分别为2.3%~3.8%、3.9%~6.9%。结果见表3。

**表3:** 血液和尿液中氟乙酸在不同添加水平下的回收率、日内精密度和日间精密度(*n*=6)

Added/ (μg/L)	Blood				Urine		
	Rec./ %	Intra-day RSD/%	Inter-day RSD/%		Rec./ %	Intra-day RSD/%	Inter-day RSD/%
10.0	94.8	0.8	2.3		96.2	0.2	6.9
50.0	93.4	1.6	3.2		97.9	1.0	3.9
200.0	95.8	1.0	3.8		98.4	0.7	4.0

Rec.: recovery.

#### 2.4.3 基质效应

血液和尿液等生物检材成分复杂,进一步考察基质效应对于验证本研究成果的可靠性具有重要意义。本研究通过测定血液、尿液基质匹配标准溶液和标准工作溶液中氟乙酸的峰面积,获得线性范围内(0.5、1.0、5.0、20.0、100.0、200.0、500.0 μg/L)线性方程的斜率,并按如下公式计算基质效应:

ME= $\frac{slope_{M}-slope_{S}}{slope_{S}}$ ×100%

其中,slope_M_为基质匹配标准溶液曲线的斜率;slope_S_为氟乙酸标准溶液曲线的斜率。

当ME的绝对值在0%~25%范围内时,表明待测物在血液或尿液中有弱基质效应;当ME的绝对值大于25%时,表明待测物在血液或尿液中有中等强度以上的基质效应。本研究按照1.3节的方法处理血液和尿液样品,添加标准溶液后上机检测。结果显示,氟乙酸在血液和尿液中的基质效应分别为-7.4%、-3.0%, ME的绝对值均小于25%,基质效应弱,具体见表4。同时对乙腈沉淀蛋白质-RP柱固相萃取法处理的血液和尿液样品进行了基质效应考察,基质效应分别为9.0%、8.3%。与ASE-离心超滤法对比,该法不仅操作繁琐,且基质效应较强,这进一步验证了ASE在前处理方面的优势。

**表4:** 血液和尿液中氟乙酸检测的线性关系和基质效应

Matrix	t_R_/ min	Linear range/ (μg/L)	Regression equation	Correlation coefficient (r)	ME/ %
Water	5.16	0.5-500.0	y=15620.3x+3672.2	0.9995	0.0
Blood	5.15	0.5-500.0	y=14459.8x+2944.6	0.9999	-7.4
Urine	5.16	0.5-500.0	y=15152.7x+3283.2	0.9995	-3.0

*y*: peak area; *x*: mass concentration, μg/L.

此外,对比了标准溶液和血液、尿液基质标准溶液中氟乙酸的时间偏移情况。结果表明,血液和尿液基质中的氟乙酸保留时间偏差均小于1%,基质效应对保留时间影响较小,如图3。综上所述,空白血液和尿液经处理后对氟乙酸检测的影响小,ASE-离心超滤法能够满足氟乙酸定量分析的要求。

**图3:**
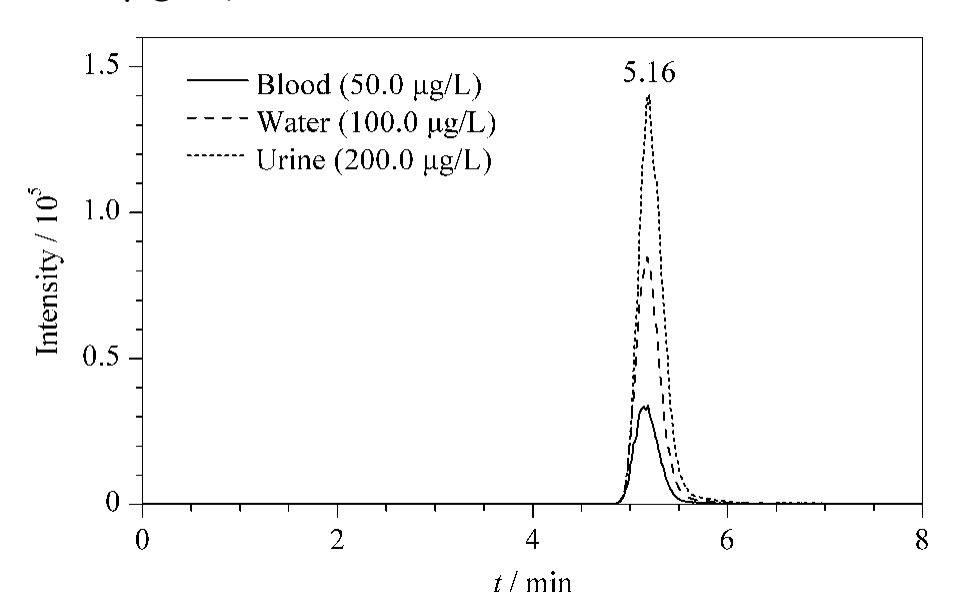
氟乙酸在不同基质中保留时间的对比

### 2.5 实际样品检测

某地发生疑似氟乙酸中毒案件,办案单位提取死者血液和尿液送检。分别取1 mL血液和尿液,按照1.3~1.5节氟乙酸检测方法进行分析,测定血液和尿液中氟乙酸的含量分别为441.1 μg/L和123.6 μg/L,结果见图4。综合法医临床检验和毒化分析结果,推断死亡原因为氟乙酸摄入过量。

**图4:**
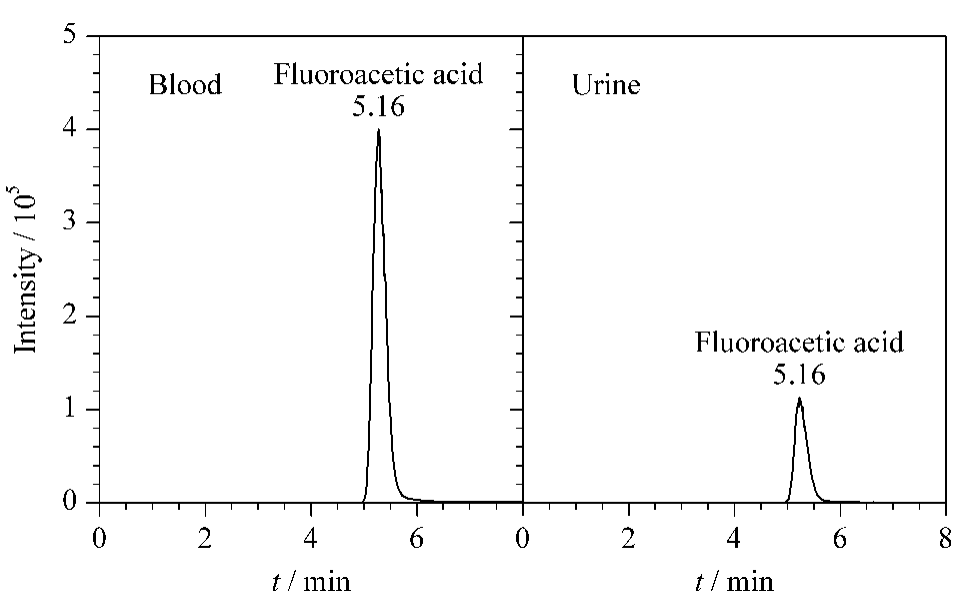
血液和尿液中氟乙酸的提取离子色谱图

在实际检测中,同时使用衍生化-GC-MS^[16]^和ASE-IC-MS测定血液和尿液中的氟乙酸。结果表明,虽然衍生化-GC-MS同样能测定出检材中含有氟乙酸,但整个检测过程耗时约4 h;而ASE-IC-MS全过程耗时小于1 h且精确度更高,检测效率显著优于传统方法。可见,ASE-IC-MS检测氟乙酸的方法准确高效,可用于实际样品的检测,能够满足办案人员对检验结果的紧迫需求,为相关案件的快速侦破提供关键信息。

## 3 结论

本研究使用非有机溶剂和快速溶剂萃取仪处理血液和尿液样品,建立了ASE-IC-MS测定人体血液、尿液中氟乙酸的方法,方法学考察表明,该法可以满足氟乙酸分析鉴定的要求,能够应用于实际案件中人体血液、尿液的快速高效检测,为公安实战中氟乙酸中毒案件的检验工作提供了新方案。

## References

[1] Leong L E X, Khan S, Davis C K, et al. J Anim Sci Biotechnol, 2017, 8(1): 1 28674607 10.1186/s40104-017-0180-6 PMC5485738

[2] Yang L, Li H F, Bai Y C, et al. Journal of Instrumental Analysis, 2021, 40(4): 495

[3] Zhao S Z, Yi X H, Shi Y Y, et al. Chinese Journal of Chromatography, 2016, 34(4): 397

[4] Zhang X Y, Zhang X Y, Cai X X, et al. Chinese Journal of Chromatography, 2018, 36(10): 979 30378356 10.3724/SP.J.1123.2018.06011

[5] Zhang X Y, Cai X X, Zhang X Y, et al. Journal of Chinese Mass Spectrometry Society, 2019, 40(1): 90

[6] Song X J, He X R, Yi M M, et al. Chinese Journal of Chromatography, 2018, 36(10): 1038 30378364 10.3724/SP.J.1123.2018.05012

[7] Jin D Q, Ding B D, Yong D M, et al. Agrochemicals, 2020, 59(1): 52

[8] Ying J B, Luo Y C. Chinese Journal of Forensic Medicine, 2010, 25(3): 181

[9] Ahmad R, Ahmad N, Shehzad A. Food Chem, 2020, 309: 125740 31711807 10.1016/j.foodchem.2019.125740

[10] Wong Y T, Law W K, Lai S S L, et al. Anal Methods, 2018, 10(28): 3514

[11] Zhang T, Wang G Q. Physical Testing and Chemical Analysis Part B: Chemical Analysis, 2022, 58(3): 270

[12] Parry E, Willison S A. Anal Methods, 2018, 10(46): 5524 10.1039/C8AY02046A PMC6309164 30598702

[13] Liu L, Hu S, Zhai S, et al. J Pharm Biomed Anal, 2018, 158: 370 29936376 10.1016/j.jpba.2018.06.028

[14] Hu G, Xu X, Zhang H, et al. Food Anal Method, 2016, 9(10): 2741

[15] Jin J H, Ye M L. Chinese Journal of Chromatography, 2016, 34(10): 960

[16] GA/T 933-2011

[17] Mu Y Q, Wu Y X, Wang X, et al. Chinese Journal of Chromatography, 2022, 40(12): 1128 36450353 10.3724/SP.J.1123.2022.01020 PMC9727743

[18] Lu S J, Yang R Q, Yu S H, et al. Journal of Instrumental Analysis, 2022, 41(2): 261

[19] Zhao H J, Liang W H, Li Z, et al. Chinese Journal of Chromatography, 2017, 35(3): 264

[20] Chen W, Liu Y, Song L, et al. Algal Res, 2020, 51: 102080

